# Circular Dichroism Study of Orexin B under Oxidative Stress Conditions

**DOI:** 10.3390/molecules28020484

**Published:** 2023-01-04

**Authors:** Martina Rotondo, Claudia Honisch, Stefano Tartaggia, Paolo Ruzza

**Affiliations:** Institute of Biomolecular Chemistry of CNR (ICB-CNR), Via F. Marzolo, 1, 35131 Padova, Italy

**Keywords:** orexin B, oxidative stress, peptide synthesis, peptide conformation, membrane mimetic environment, circular dichroism, MALDI-TOF

## Abstract

The neuropeptides orexin A and B regulate various vital functions of the body, such as sleep/wake states, metabolism, and energy homeostasis. A loss of their physiological activity, with reduced ability to recognize their receptors, is suspected to be associated with oxidative stress conditions. These are related to excessive presence of reactive oxygen and nitrogen species, as well as of reactive lipoxidation byproducts. With the aim of evaluating the effects of oxidative stress on the secondary structure of orexin peptides, orexin B was synthesized and characterized by circular dichroism spectroscopy under different conditions. In aqueous solution it presents an unordered conformation, while in a membrane mimetic environment it assumes a helical structure. The effects of oxidative stress were evaluated exposing it to both oxygen and nitrogen radicals as well as to lipoxidation byproducts. The results showed that ROS, but not NRS, induced appreciable conformational changes, and only in the membrane mimetic environment. Lipoxidation byproducts, instead, led to secondary structure modifications much more evident than those induced by the direct action of ROS and RNS, and in both analyzed media. Additionally, MALDI-TOF analyses detected mass variations in the peptide attributable to oxidation of the C-terminal Met residue and deamination of asparagine in the Asn–His sequence. Taken together, all these data seem to confirm the involvement of oxidative processes in dysfunctions of the orexinergic system.

## 1. Introduction

**Figure 1:**
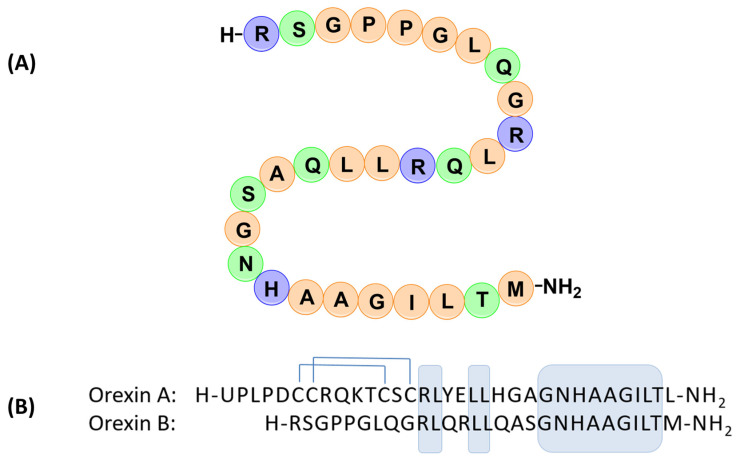
(**A**) Amino acid sequence of human orexin B peptide, and (**B**) sequences comparison of orexin A and orexin B peptides. The one-letter code of the first N-terminal residue of orexin-A (U) represents pyroglutamic acid. The C-termini of both orexins are amidated (–NH_2_). Two intramolecular disulfide bonds (C^6^–C^12^ and C^7^–C^14^) are shown as lines. The residues, identical in both orexins, are boxed in shading.

Orexin A (OXA) and orexin B (OXB), also known as hypocretin-1 and hypocretin-2, respectively, are two neuropeptides synthesized in the lateral hypothalamus, deriving from the same precursor prepro-orexin [1,2]. Both peptides are recognized by two specific receptors called orexin Receptors 1 and 2 (OX1R and OX2R). While OX1R selectively recognizes orexin A, OX2R is activated by both peptides non-selectively [1]. The two peptides exhibit a sequence homology of 46% in the C-terminal region (Figure 1B) [1,3], suggesting that this region is important for receptor recognition and their biological activity [4]. In particular, the Arg^10^-Met^28^ sequence of orexin B was identified as the shortest portion of peptides recognized by both orexin receptors [5].

Orexin B (Figure 1A) is a linear, C-terminal-amidated peptide consisting of 28 amino acids that in a membrane mimetic environment adopts a helical secondary structure [6,7]. Previously reported NMR and computational studies have detected the presence of two α-helix segments. The first helix spans from Leu^7^ to Ser^18^ residues, while the second one goes from the Ala^23^ to the Met^28^ residues, oriented about 60–80° relative to each other [6,7]. Moreover, the presence of two proline residues in the N-terminal portion of orexin B (Pro^4^–Pro^5^) induces the formation of a β-turn secondary structure [8].

Orexin A is a 33 amino acid amidated peptide containing two intramolecular disulfide bridges (Cys^6^–Cys^12^, Cys^7^–Cys^14^) and an N-terminal pyroglutamoyl residue. The NMR-structure in membrane mimetic environment is characterized by the presence of three helical sections (helix I: Leu^16^–Ala^23^, helix II: Asn^25^–Thr^32^, and helix III: Cys^6^–Gln^9^) [3]. 

Neurons expressing the peptides orexin A and B are involved in the transmission of signals related to environmental, physiological, and emotional stimuli. Furthermore, they are involved in the regulation of several vital functions of the body, including sleep/wake rhythms [9], energy homeostasis and metabolism [10,11], reward systems [12], cognition, and mood [13]. Consequently, dysfunctions of the orexinergic system can underlie several pathological conditions. For example, a selective loss of orexin neurons has been found in narcolepsy [9], while an orexin deficiency is associated with the onset of obesity. Indeed, the levels of orexin are strictly correlated to spontaneous physical activity. High levels of these peptides increase spontaneous physical activity promoting resistance to obesity, while low levels reduce physical activity with consequent weight gain [11]. A deficiency of orexin has been associated with learning and memory impairments and depression [14].

A loss of activity of orexin A and B peptides usually occurs under oxidative stress conditions. These conditions are caused by an imbalance between the production and accumulation of reactive species or free radicals in cells and tissues and the ability of the biological system to eliminate these products [15]. If the production of reactive species is excessive, or if adaptive systems are compromised, there is an accumulation of oxidizing substances within cells and tissues. Among the various classes of oxidizing species, the most abundant are reactive oxygen (ROS) and nitrogen (RNS) species. These molecules are responsible for the oxidation of biological macromolecules, such as proteins, lipids, and nucleic acids, which can lead to the onset of serious damage to the organism, inducing different pathologies [16,17].

ROSs are byproducts of aerobic metabolism, which takes place during the mitochondrial respiratory chain [18], and include superoxide anion (O_2_^•−^), hydrogen peroxide (H_2_O_2_), and hydroxyl radical (HO^•^) [19]. These molecules are associated with oxidative stress, though over the past two decades it has been observed that ROS also play an important role as signaling molecules regulating biological and physiological processes [20].

Similarly, nitrosative stress concerns the chemistry of reactive nitrogen-based species that include nitrogen oxide (NO^•^) and nitrogen dioxide (NO_2_^•^) as well as nonradical species such as peroxynitrite ion (ONOO^−^) and nitrosamine [21]. 

Reactive species can react with peptides and proteins either to side chains of individual amino acid residues exposed to the solvent or their backbone causing breakages, formation of inter- or intramolecular bonds, or peroxides that allow the propagation of oxidative damage [22]. All these damages can lead to protein unfolding or the formation of protein aggregates, altering their recognition by other molecules or receptors and modifying their function [23].

Oxidative stress can also induce lipid oxidation, giving rise to reactive electrophilic products, such as small aldehydes and α,β-unsaturated carbonyl fragments capable of forming covalent adducts with a nucleophilic moiety of proteins and nucleic acids [24]. This phenomenon is called lipoxidation and the adducts obtained are called advanced lipoxidation end products (ALEs) by analogy with the formation of advanced glycoxidation end products (AGEs) from oxidized sugars. Lipoxidation products, interacting with proteins, can alter their structure and, for larger and longer chain adducts, can introduce relatively hydrophobic and voluminous species. This process can cause enzyme inactivation, interactions with altered macromolecules, unfolding and increased proteasome degradation, but also upregulation of cellular defense responses. Furthermore, protein modification can induce an increase in β-strand conformation, which tends to afford amyloid-like structures and oligomerization or unfolding and aggregation [25,26]. 

With the aim of evaluating the effects of oxidative stress on the secondary structure of orexin B, we synthesized the orexin B peptide and characterized its secondary structure in an aqueous and membrane mimetic environment. The effects of ROS and RNS as well as of byproducts of lipoxidation were evaluated by both circular dichroism and MALDI-TOF mass spectroscopy. Results found that the secondary structure of orexin B is sensitive to the action of ROS, while no evident alterations are observed due to the effect of RNS. On the contrary, the action of the byproducts of lipid oxidation substantially modifies the conformation of the peptide itself, suggesting a predominant role for this mechanism in determining the loss of activity of orexin B.

## 2. Results

### 2.1. Peptide Synthesis

Orexin B peptide was synthesized by manual solid-phase peptide synthesis (SPPS), using Fmoc chemistry in 0.1 mmolar scale starting from Fmoc–MBHA Rink amide resin. A threefold molar excess (0.4 mmol) of Fmoc amino acids was used for each coupling step, using HBTU/HOBt as coupling reagents in the presence of DIPEA. The Fmoc group was removed using a 20% *v*/*v* solution of piperidine in DMF up to the His^21^ residue. Successively, the Fmoc removal strategy was changed and from the residue Asn^20^, a 5% *w*/*v* solution of piperazine in DMF was used to avoid the aggregation of the growing peptide that can interfere with the removal of the Fmoc group. Indeed, piperazine, acting as a chaotropic molecule, prevents this aggregation which can lead to the formation of truncated or deleted sequences [27]. However, after the detachment of peptide from the resin by TFA treatment, the presence of various truncated sequences was detected, which lowered the yield and hindered peptide purification.

To increase the synthetic yield, the strategy for the synthesis of orexin B was modified, and starting from the coupling of the His^21^ residue, HATU was used as coupling reagent instead of HBTU, exploiting the superior properties of HATU in activating the carboxyl group and in facilitating the subsequent condensation reaction [28]. In addition, double coupling procedures in the condensation of the amino acids Leu^14^, Arg^13^, Arg^11^, Gln^9^, and Pro^4^ were introduced. Moreover, also the Fmoc removal strategy was further modified. The 5% *w*/*v* solution of piperazine in DMF was added to 2% DBU. Using all these precautions, a sufficient pure raw peptide was obtained after treatment with TFA that was purified by preparative RP-HPLC according to the procedure described in the Section 4.

### 2.2. Conformational Studies

The secondary structure of orexin B was evaluated by circular dichroism spectroscopy, both in aqueous and in membrane mimetic environment. 

**Figure 2:**
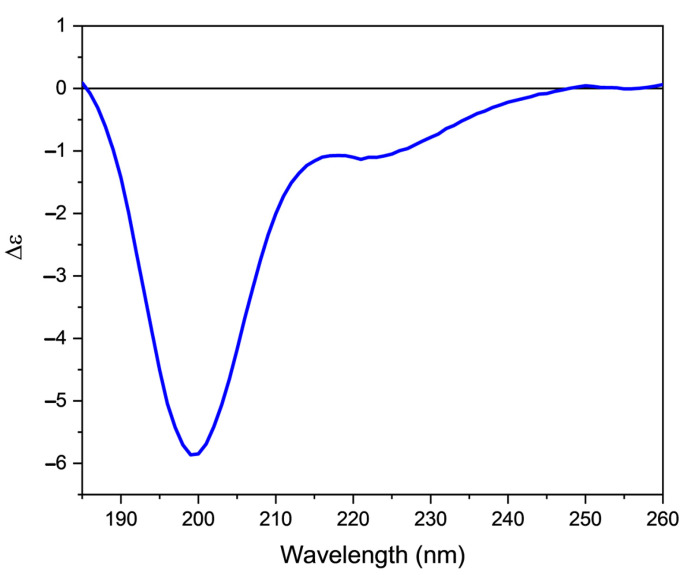
Far-UV CD spectrum of orexin B (32 µM) in 20 mM phosphate buffer, pH 7.4. Spectrum was recorded at 25 °C using a Jasco J-1500 spectropolarimeter.

The CD spectrum of orexin B (Figure 2) in 20 mM phosphate buffer, pH 7.4, is characterized by the presence of a negative band at 199 nm accompanied by a shoulder at 225 nm. This spectrum is characteristic of an unordered structure, as confirmed by the analysis of the secondary structure content calculated using the CONTINLL algorithm [29] implemented in CDApps software [30] (Table S1 in Supplementary Materials). Using this algorithm, a percentage greater than 40% was determined for the unordered structure, while an equal percentage of turns and β-sheet secondary structure (about 25% for each structure) was determined.

The absence of an ordered structure was confirmed by the acquisition of CD spectra of orexin B in phosphate buffer at different temperatures (Figure S1 in Supplementary Materials). A progressive decrease in the intensity of the dichroic signal at 199 nm, accompanied by the impossibility of obtaining a significant melting curve (insert in Figure S1) characterized the obtained results.

**Figure 3:**
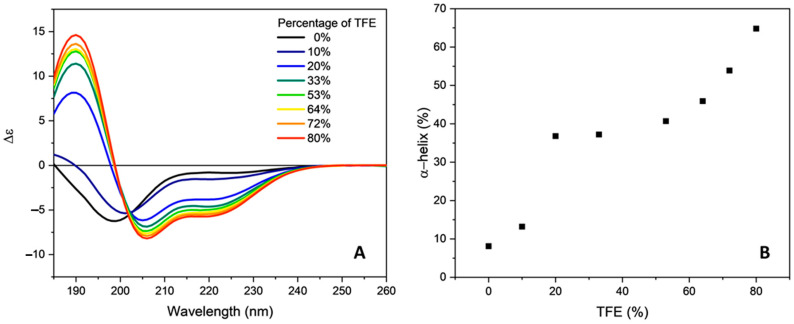
Influence of TFE on the secondary structure of orexin B. (**A**) Far-UV CD spectra of orexin B (32 µM) in presence of different amount of TFE (indicated as %). Spectra were recorded at 25 °C using a Jasco J-1500 spectropolarimeter. (**B**) Secondary structure content (% of α-helix) as a function of the percentage of TFE.

The addition of increasing amounts of 2,2,2-trifluoroethanol (TFE), an alcohol that mimics a membrane mimetic environment with helicogenic properties [31], induced a progressive change in the dichroic spectrum with the appearance of an intense positive band at 190 nm and two negative bands at 205 nm and 220 nm (Figure 3A), characteristic of an α-helix structure. This conformational transition agrees with reported NMR studies in a membrane mimetic environment [6,7].

In addition, the appearance of an isodichroic point at 202 nm at percentages of TFE equal to or greater than 20% indicates the presence of an equilibrium between two species: the unordered and the α-helix peptide conformations. The content in α-helix, determined by the CONTINLL algorithm, remains constant at about 40% in the range of TFE concentration between 20 to 60%, and then progressively increases up to about 65% with the increasing TFE content (Figure 3B).

Two mechanisms have been proposed to explain the induction of a helical structure by TFE. The stabilization of this secondary structure was attributed to a positive effect on the H-bonding interaction. This alcohol does not compete for H-bonding as effectively as water, and lowers the dielectric constant appreciably, strengthening intramolecular H-bonds [32]. Moreover, the transfer of a peptide to TFE is thermodynamically unfavored [33]. Since helix formation shields the peptide backbone from solvent by the action of side chains, the stabilization of an α-helix by TFE can also be considered a solvophobic effect [34].

**Figure 4:**
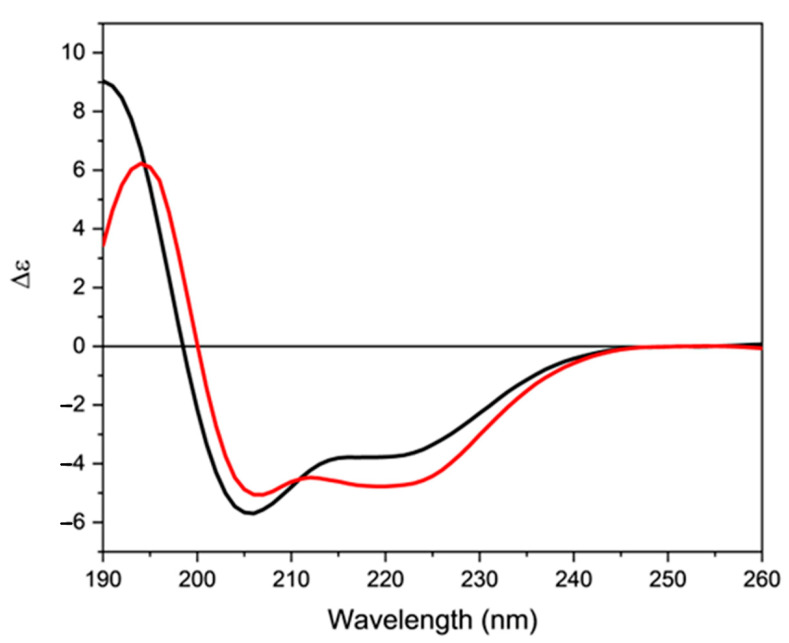
Far-UV CD spectra of orexin B in presence of either micellar SDS solution (black line) or DMPG/DMPC 1:3 m/m SUVs (red line). Peptide concentration was 34 µM. Spectra were recorded at 25 °C using a Jasco J-1500 spectropolarimeter.

In the presence of a micellar sodium dodecyl sulfate (SDS) solution (Figure 4, black line), the CD spectrum closely resembles that determined in presence of 80% of TFE. However, the intensity of both positive and negative bands was decreased, suggesting a lower content in ordered structure. Indeed, the percentage of α-helix determined using the CONTINLL algorithm decreased at about 45% (Table S1 in Supplementary Materials).

On the contrary, in the presence of 1,2-dimyristoyl-sn-glycero-3-phosphorylglycerol/1,2-dimyristoyl-sn-glycero-3-phosphocholine small unilamellar vesicles (DMPG/DMPC SUVs) (Figure 4, red line), the CD spectrum of orexin B is characterized by a change in the intensity and shape of the two negative bands. Indeed, a shift to higher wavelength (195 nm) of the positive band was accompanied by a decrease in its intensity compared to the values determined in presence of TFE. In particular, in presence of either TFE or SDS the negative band at 220 nm was less evident with a θ_MRW,205_/θ_MRW,220_ ratios of 0.70 and 0.66, respectively. On the contrary, in the presence of DMPG/DMPC SUVs the 220 nm band intensity is comparable to that of the 206 nm band and the θ_MRW,205_/θ_MRW,220_ ratio is 0.94 with a percentage of α-helix of about 30%. This behavior suggests a possible propensity of the two helix segments present in the orexin B structure to adopt a different spatial arrangement in the presence of unilamellar vesicles.

### 2.3. Effect of Oxidative Stress on the Secondary Structure of Orexin-B

The influence of reactive species on the secondary structure of orexin B was evaluated in different conditions. Initially, aqueous peptide solutions in phosphate buffer and in the presence of phospholipids were irradiated for increasing times with a UV-C lamp (254 nm) placing the cuvette containing the peptide solution in front of the lamp itself. The UV radiation leads to the formation of ROS mainly due to water photolysis, according to Scheme S1 reported in Supplementary Materials. In previous works, indeed, we demonstrated that UV-C irradiation induced the formation of ROSs in aqueous media and that their amount is proportional to the exposure time. Moreover, we also demonstrated that these reactive species induced protein conformational changes that can be used to evaluate the influence of ligands and/or environment on protein stability [35,36].

**Figure 5:**
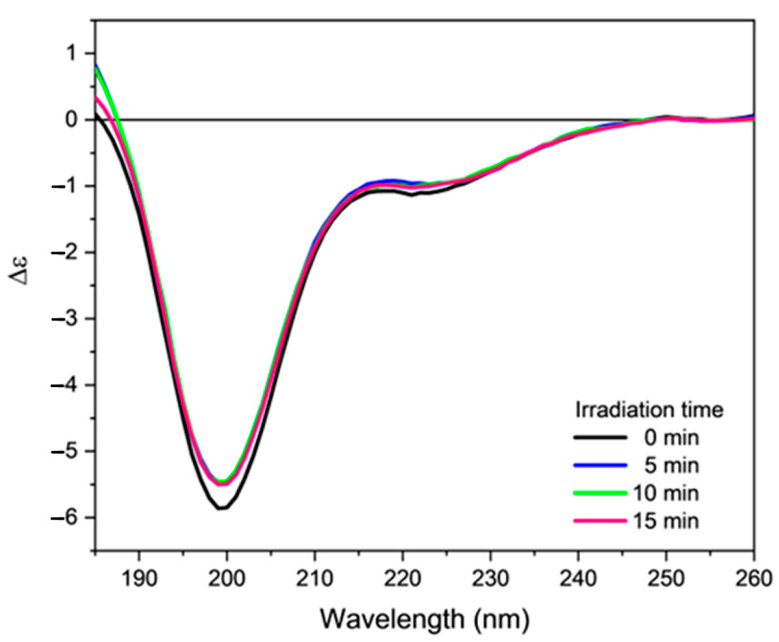
Far-UV CD spectra of orexin B (34 µM) in 20 mM phosphate buffer, pH = 7.4 irradiated for increasing time (indicated) with a UV-C lamp at room temperature. Spectra were recorded at 25 °C using a Jasco J-1500 spectropolarimeter.

The CD spectra of orexin B in phosphate buffer irradiated for increasing times are reported in Figure 5. The collected spectra are almost superimposable, indicating that irradiation has little or no effect on the secondary structure of orexin B (compare Tables S1 and S2 in Supplementary Materials). The MALDI-TOF analysis of buffer peptide solution after irradiation revealed the presence of a small amount of a peptide having a m/z value 16 units higher than orexin B, most likely due to the formation of methionine sulfoxide (Figure S2 in Supplementary Materials). This C-terminal residue is the most sensitive and the most exposed to oxidizing action by ROS; however, its oxidation is not sufficient to induce an evident change in the CD spectrum of the investigated peptide in phosphate buffer solution, where the peptide exhibited an unordered structure.

**Figure 6:**
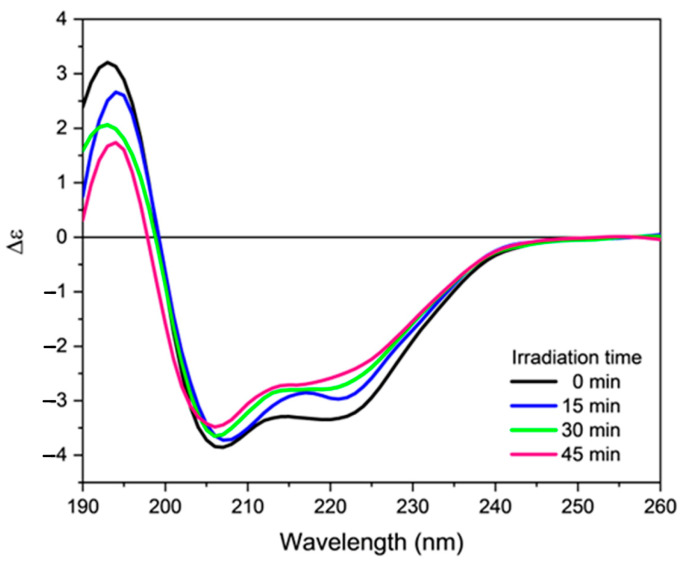
Far-UV CD spectra of orexin B (34 µM) in DMPG/DMPC 1:3 m/m SUVs, irradiated for increasing time (indicated) with a UV-C lamp at room temperature. Spectra were recorded at 25 °C using a Jasco J-1500 spectropolarimeter.

On the contrary, in the presence of phospholipids (Figure 6), where the peptide adopts a helical structure, the exposure to UV light leads to a decrease in the intensity of both previously described positive and negative bands. This event is accompanied by a decrease in the α-helix content with a corresponding increase in the β-sheet content (Table S2 in Supplementary Materials). The effects of UV irradiation are very evident on the intensity of the positive band at about 195 nm and on the intensity of the negative band at 220 nm, while the negative band at 205 nm is scarcely affected by exposure to UV-C light. Moreover, in presence of SUVs, the MALDI-TOF spectrum showed the presence of a molecule having an m/z value 16 units higher than orexin B attributable to the oxidation of the C-terminal Met residue (Figure S3 in Supplementary Materials). As mentioned above, the presence of methionine sulfoxide scarcely affects the secondary structure of orexin B in buffer. However, in the presence of DMPG/DMPC SUVs its formation is detrimental to the ordered structure adopted by the peptide in physiological conditions. These observations suggest that the oxidation of methionine is sufficient to disrupt the ordered structure of orexin B, which may result in a loss of activity. 

**Figure 7:**
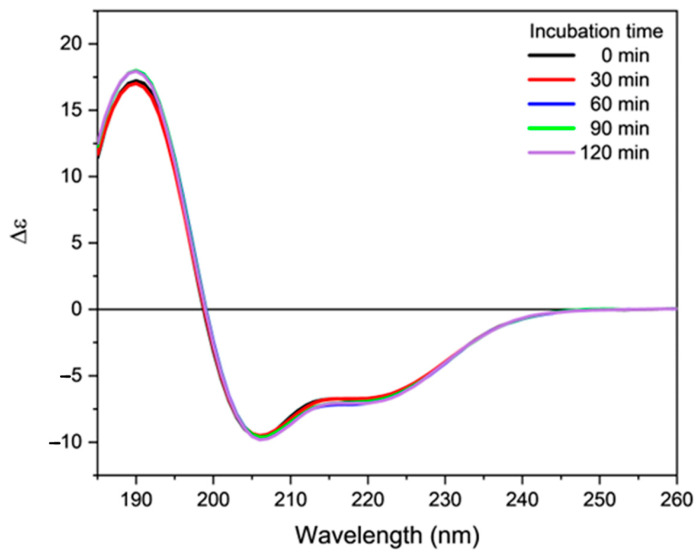
Far-UV CD spectra of orexin B (34 µM) in 80% TFE added of 20 eq. of DEA NONOate. The spectra were acquired at increasing time of incubation (indicated) at 25 °C using a Jasco J-1500 spectropolarimeter.

Successively, the effects of nitrogen radicals on the secondary structure of orexin B were evaluated. For this purpose, phosphate buffer and 80% TFE peptide solutions were treated with 20 equivalents of DEA-NONOate, a source of nitrosyl radicals NO^•^ according to the mechanism reported in Scheme S2 in Supplementary Materials. Surprisingly, the dichroic spectra recorded at different incubation times do not show any substantial differences in the intensity and shape of the spectra (Figure 7 and Figure S4 in Supplementary Materials). This suggests the absence of any significant action by the RNS towards the secondary structure of orexin B. 

Surprisingly, the MALDI-TOF spectra of orexin B incubated with DEA-NONOate show the presence of a peak at m/z value 17 units lower than that orexin B. This peak is more evident in the analysis of the buffer peptide solution than that in the 80% TFE peptide solution (Figures S5 and S6 in Supplementary Materials). A possible cause of the presence of this peak is the deamination of the Asn residue with the formation of a succinimide intermediate according to the Scheme S3 in Supplementary Materials. This occurrence is accelerated when a His residue (sequence Asn^20^-His^21^) is present in the C-terminal position of the asparagine [37]. Future studies are necessary to confirm this side-reaction and its mechanism in in vitro conditions and above all to determine the presence of this secondary reaction in vivo at the level of orexinergic neurons.

Finally, the action of two reactive byproducts deriving from lipid oxidation on the secondary structure of orexin B was evaluated. For this purpose, the buffer and 80% TFE orexin B solutions were incubated with either glyoxal or pyruvic aldehyde, two byproducts formed by both lipid and sugar oxidation. The presence of two aldehydric groups allows for the formation of more stable adducts in aqueous solution that can be efficiently detected by mass spectroscopy (Figures S7 and S8, and Scheme S4 in Supplementary Materials). 

**Figure 8:**
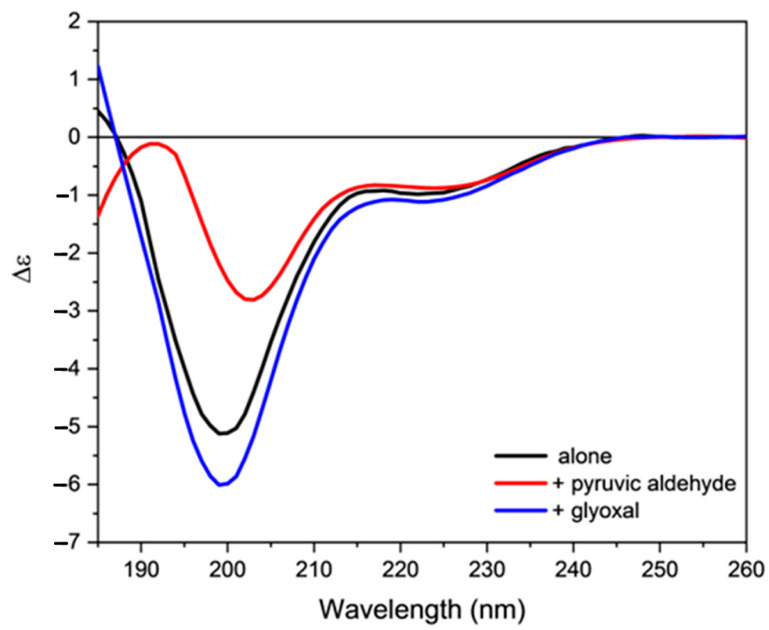
Far-UV CD spectra of orexin B (34 µM) in 20 mM phosphate buffer, pH = 7.4, added with either pyruvic aldehyde or glyoxal. The spectra were acquired at 25 °C after 30 min of incubation at 25 °C using a Jasco J-1500 spectropolarimeter.

**Figure 9:**
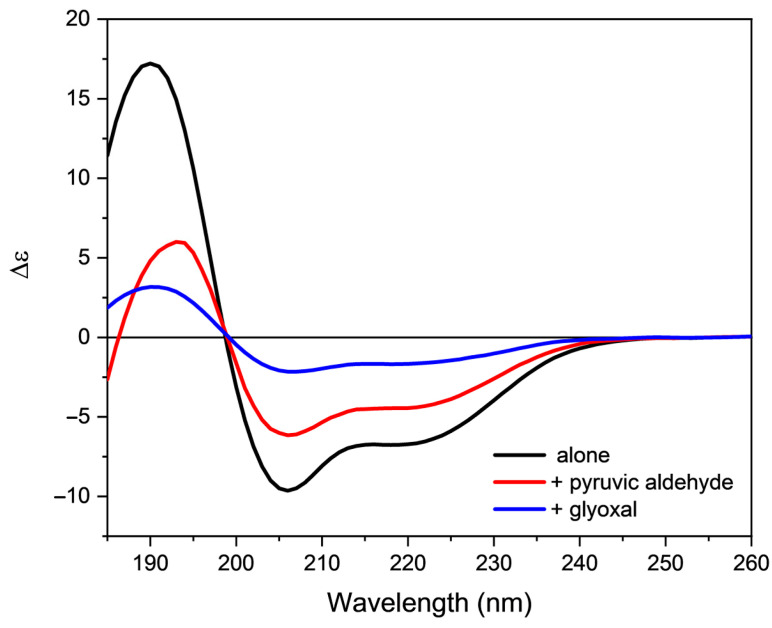
Far-UV CD spectra of orexin B (34 µM) in 80% TFE added of either pyruvic aldehyde or glyoxal. The spectra were acquired at 25 °C after 30 min of incubation at 25 °C using a Jasco J-1500 spectropolarimeter.

The incubation with the two reactive species induces an evident modification in the dichroic signal of orexin B in both the analyzed media. In particular, a strong decrease in the amount of ordered structure in membrane mimetic environment was detected (Figure 8 and Figure 9). These results clearly indicate that lipoxidation byproducts alter the secondary structure of peptides and proteins, with effects that are concentration dependent.

## 3. Conclusions

An increasing number of people suffer from neurodegenerative diseases, which are characterized by progressive loss of neurons, including those belonging to the orexinergic system. An important role in the development of these diseases is played by the oxidative stress and in particular by the overproduction of reactive oxygen species. These molecules not only react directly with biological targets such as peptides and proteins but also cause lipid oxidation, which leads to the formation of many reactive species.

The results reported in this paper clearly indicate that lipoxidation byproducts can interact with orexin B, inducing covalent modification and conformational changes much more evident than those induced by the direct action of ROS and RNS, suggesting a prominent role for these species in modifying the biological activity of the neuropeptide itself. Future work is necessary to confirm the involvement of lipoxidation byproducts in the loss of activity of the orexin B peptide and in the onset of related neurodegenerative diseases.

## 4. Material and Methods

### 4.1. Peptide Synthesis

Fmoc-protected amino acids, Fmoc–Rink amide MBHA, and coupling reagents (HBTU, HOBt, and HATU) were purchased from Irish Biotech (Marktredwitz, Deutschland). DMF, DIPEA, and solvents were obtained from Sigma-Aldrich (Milan, Italy). Peptides were synthesized by manual solid phase using Fmoc chemistry in a 0.1 mmol scale. HBTU/HOBt and HATU activation employed a threefold molar excess (0.4 mmol) of Fmoc amino acids in DMF for each coupling cycle unless otherwise stated. Coupling times were 45 min. Fmoc deprotection was performed with 20% *v*/*v* piperidine in DMF up to His^21^ and then using a 5% *w*/*v* piperazine solution in DMF in presence or not of 2% DBU. The cleavage from the resin was performed by treatment with TFA-triisopropylsilane-H_2_O (95:3:2 *v*/*v*). The peptide was purified by preparative reverse-phase HPLC using a Shimadzu LC-8 (Shimadzu, Kyoto, Japan) system with a Vydac 218TP1022, 10μ, 250 × 22 mm column (Dionex, Sunnyvale, CA). The column was perfused at a flow rate of 12 mL/min with a mobile phase containing solvent A (0.05% TFA in water) and a linear gradient of solvent B (0.05% TFA in acetonitrile/water, 9:1 by vol.). The fractions containing the desired product were collected and lyophilized to constant weight. Analytical HPLC was performed on a Shimadzu LC-10 instrument fitted with a Jupiter C18, 10 μ, 250 × 4.6 mm column (Phenomenex, Torrance, CA) using the above solvent system (solvents A and B), flow rate of 1 mL/min, detection at 216 nm. The molecular weight of the compound was determined by LC-ESI-MS on an Agilent Infinity II system. 

### 4.2. Circular Dichroism Studies

The CD spectra were recorded on a Jasco J-1500 dichrograph using the Spectra Manager software (Jasco, Tokyo, Japan). CD spectra were recorded in the 185–260 nm range, with data pitch 1 nm, scanning speed 50 nm/min, digital integration time 1 sec, and bandwidth 1 nm. The recorded spectra are the average of 4 scans, except in the presence of SUVs, where 9 scans were acquired. The quartz cell used has an optical path of 0.1 cm (Hellma Analytics, Milan, Italy). The data obtained were processed using the CDApps [30] and Origin2018 (OriginLab Corporation, Northampton, MA, USA) software. Initially, a stock water solution of the orexin B (0.5 mg/mL) was prepared. The dilution with appropriate solvents provides peptide solutions for the CD analysis (0.1 mg/mL). Melting curves were recorded in the range 185–260 nm in a temperature range of 5–90 °C, every 5 °C, with a temperature gradient of 2 °C/min.

### 4.3. Induction of Oxidative Stress

Oxidative stress was initially induced by irradiation of the peptide solutions (in phosphate buffer and DMPG/DMPC SUVs) by an UV-C lamp (254 nm) placing the quartz cell 15 cm from the light source for increasing time and recording the CD spectra after each treatment.

The effect of RNS was evaluated adding to the phosphate buffer and DMPG/DMPC SUVs peptide solutions 20 eq. of DEA-NONOate and CD spectra were recorded at increasing incubation times.

The effect of glyoxal and pyruvic aldehyde on the secondary structure of orexin B was evaluated by adding 5 eq. of reagent to the phosphate buffer and DMPG/DMPC SUV peptide solutions: solutions were incubated for 30 min before recording the CD spectra.

## Data Availability

The data presented in this study are available on request from the corresponding author.

## Supplementary Materials

The following supporting information can be downloaded at: https://www.mdpi.com/article/10.3390/molecules28020484/s1. Table S1: Analysis of the orexin B secondary structure content in the investigated solvent environments.; Table S2: Analysis of the orexin B secondary structure content when irradiated and with DEA NONOate; Figure S1: CD melting plot of orexin B in phosphate buffer; Figure S2: MALDI-TOF MS analysis of orexin B irradiated in phosphate buffer; Figure S3: MALDI-TOF MS analysis of orexin B irradiated in phospholipids; Figure S4: CD spectra of orexin B in phosphate buffer in presence of DEA NONOate at increasing incubation times; Figure S5: MALDI-TOF MS analysis of orexin B in presence of DEA NONOate in phosphate buffer; Figure S6: MALDI-TOF MS analysis of orexin B in presence of DEA NONOate in 80% TFE; Figure S7: MALDI-TOF analysis of orexin B in presence of glyoxal in phosphate buffer; Figure S8: MALDI-TOF MS analysis of orexin B in presence of pyruvic aldehyde in phosphate buffer. Scheme S1: Mechanism of ROS generation in aqueous medium due to UV irradiation; Scheme S2: Mechanism of NO· radical formation due to DEA NONOate; Scheme S3: Mechanism of Asn residue deamination with the formation of a succinimide intermediate; Scheme S4: Mechanism of stable adducts formation among glyoxal or pyruvic aldehyde with amino acid residues.
