# Supplementary material for: Circular Dichroism Study of Orexin B under Oxidative Stress Conditions
Source: Molecules. 2023 Jan 4;28(2):484. doi: 10.3390/molecules28020484 (PMC9863598; doi:10.3390/molecules28020484)
Supplement: Supplementary file 1 [file molecules-28-00484-s001.zip › molecules-2094962-supplementary.pdf]

# Circular Dichroism Study of Orexin B under Oxidative Stress Conditions

**Martina Rotondo[1,#], Claudia Honisch[1,#], Stefano Tartaggia[1], Paolo Ruzza[1,*]**

[1] Institute of Biomolecular Chemistry of CNR (ICB-CNR), Via F. Marzolo, 1 , 35131 Padova (IT) ; martina.rotondo.1@studenti.unipd.it (M.R.); claudia.honisch@icb.cnr.it (C.H.); stefano.tartaggia@cnr.it (S.T.); paolo.ruzza@cnr.it (P.R.)

\# These authors contributed equally to this work

* Correspondence: paolo.ruzza@cnr.it; Tel.: +39 049 827 5282

## Supplementary Materials

**Table S1.** *Analysis of the orexin B secondary structure content in the investigated solvent environments.*

| | α-helix | β-strand | Turns | Unordered | $\theta_{MRW,205}$ | $\theta_{MRW,220}$ | Ratio $\theta_{MRW,205}/\theta_{MRW,220}$ |
|---|---|---|---|---|---|---|---|
| Buffer | 9.3% | 24.3% | 24.2% | 42.2% | -1062.95 | -289.80 | n.d. |
| TFE 20% | 36.8% | 17.9% | 21% | 24.3% | -1893.02 | -1162.31 | 0.61 |
| TFE 80% | 64.8% | 2.6% | 13.9% | 18.7% | -2885.51 | -2027.57 | 0.70 |
| SDS | 43.4% | 11.5% | 17.7% | 27.4% | -1746.05 | -1147.82 | 0.66 |
| DMPG/DMPC | 31% | 14.8% | 22.4% | 31.8% | -1484.19 | -1387.94 | 0.94 |

*n.d. : non determined*

**Table S2.** *Analysis of the orexin B secondary structure content when irradiated and with DEA NONO-Ate.*

| | Orexin B in | α-helix | β-strand | Turns | Unordered |
|---|---|---|---|---|---|
| **phosphate buffer, irradiated** | 0 min | 9.3% | 24.3% | 24.2% | 42.2% |
| | 5 min | 8.5% | 27.5% | 23.5% | 40.5% |
| | 10 min | 8.5% | 28.1% | 23.2% | 40.2% |
| | 15 min | 8.7% | 27.2% | 23.7% | 40.4% |
| **DMPG/DMPC, irradiated** | 0 min | 31% | 14.8% | 22.4% | 31.8% |
| | 15 min | 36.5% | 6.7% | 20.6% | 36.2% |
| | 30 min | 27.1% | 19.2% | 22.8% | 30.9% |
| | 45 min | 26.6% | 18.2% | 24.2% | 31% |
| **DEA NONO-Ate in phospate buffer** | 0 min | 9.3% | 24.3% | 24.2% | 42.2% |
| | 30 min | 8.2% | 27.3% | 23.5% | 40.9% |
| | 60 min | 7.3% | 28.7% | 22.8% | 41.1% |
| | 90 min | 7.9% | 27.5% | 23% | 41.7% |
| | 120 min | 8.5% | 26.8% | 23.5% | 41.2% |
| **DEA NONO-Ate in TFE** | 0 min | 64.8% | 2.6% | 13.9% | 18.7% |
| | 30 min | 66.8% | 2.7% | 8.9% | 21.6% |
| | 60 min | 67.3% | 4.7% | 6.8% | 21.2% |
| | 90 min | 64.9% | 4.2% | 7.8% | 23.1% |
| | 120 min | 64.8% | 4.2% | 7.8% | 23.2% |

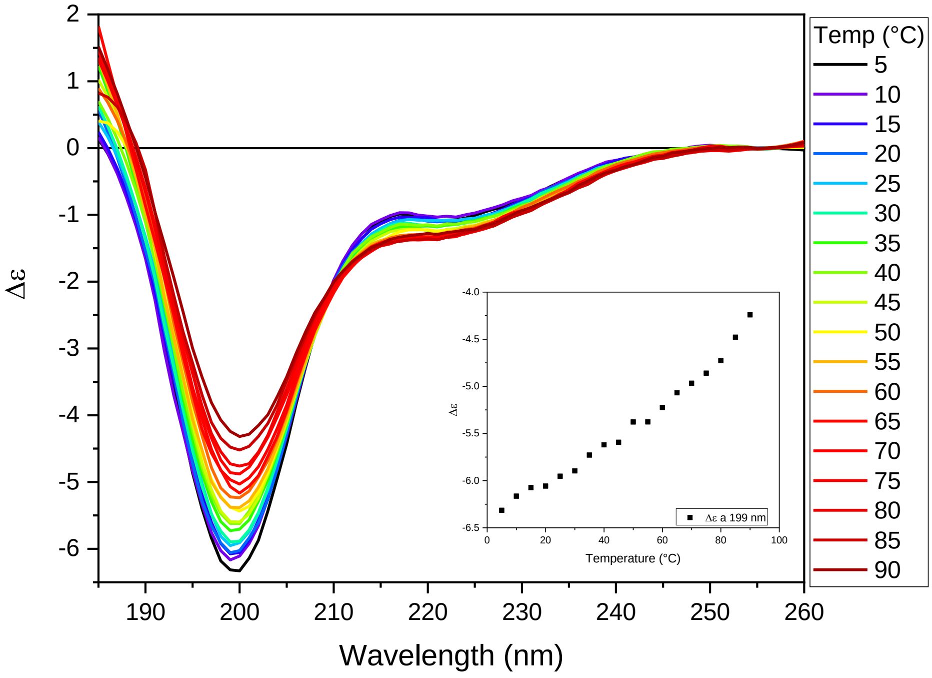


**Figure S1.** *CD melting plot of orexin B (34 µM in phosphate buffer). Spectra were recorded on a Jasco J-1500 dichrograph in the 185-260 nm range in the 5-90°C temperature range every 5°C, with a temperature gradient of 2°C/min.* ***Insert****: plot of Δε at 199 nm versus temperature.*

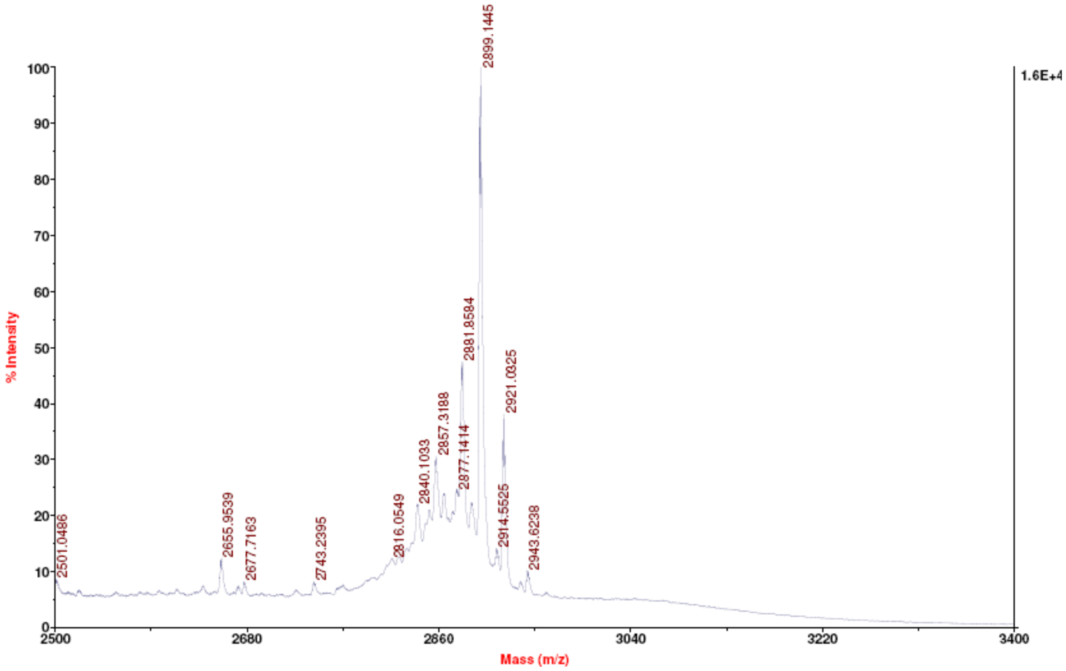


**Figure S2.** *MALDI-TOF MS analysis of orexin B in phosphate buffer, irradiated. Matrix: α-Cyano-4-hydroxycinnamic acid in 9: 1 = MeCN: $H_2O$ + 0.1% TFA. Orexin B [M+H$^+$] =2899 m/z; Orexin B Met(O) [M+H$^+$] =2915 m/z.*

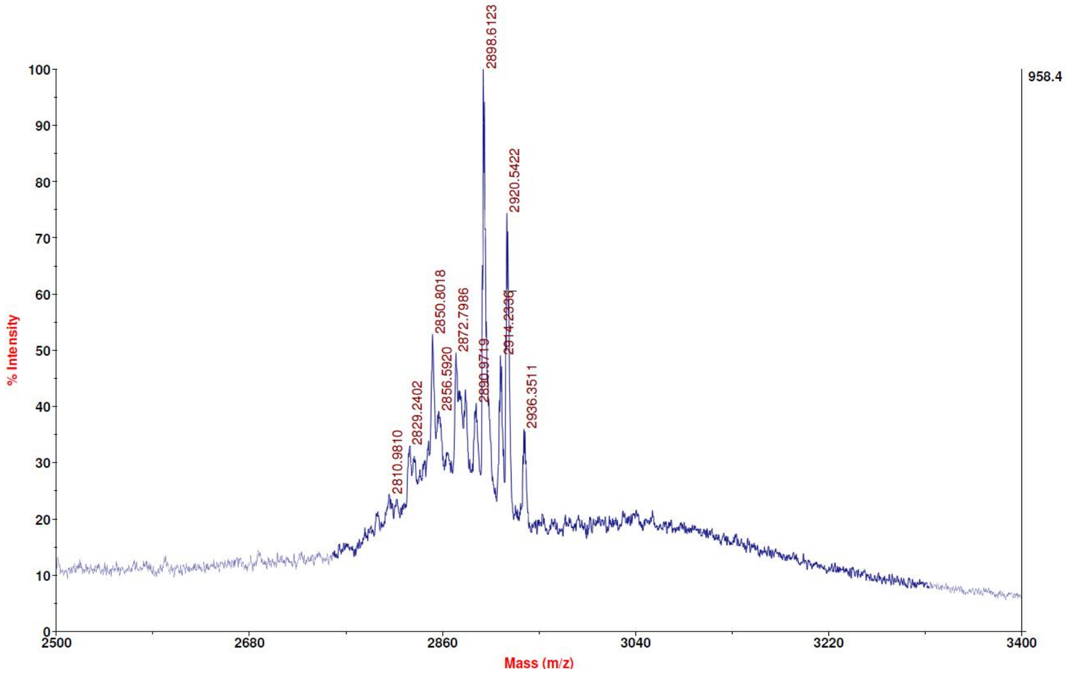


**Figure S3.** *Orexin B in phospholipids, irradiated. Matrix: α-Cyano-4-hydroxycinnamic acid in 9: 1 = MeCN: $H_2O$ + 0.1% TFA. Orexin B [M+H$^+$] =2899 m/z; Orexin B Met(O) [M+H$^+$] =2915 m/z.*

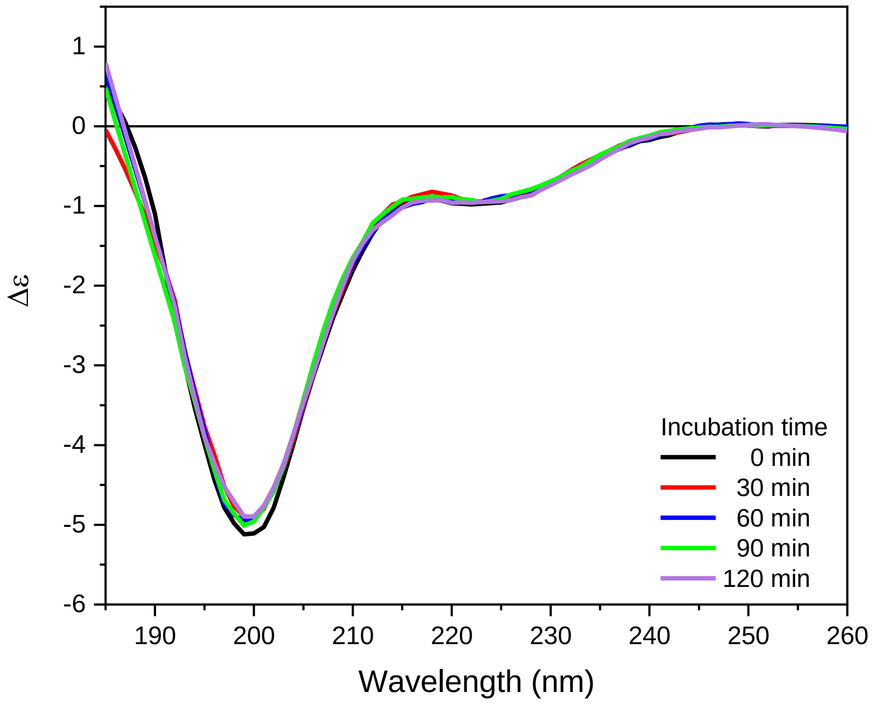


**Figure S4.** *Far-UV CD of orexin B (34 μM) in 20 mM phosphate buffer, added of 20 eq. of DEA NONOate. Spectra were recorded on a Jasco J-1500 dichrograph at increasing incubation times (indicated).*

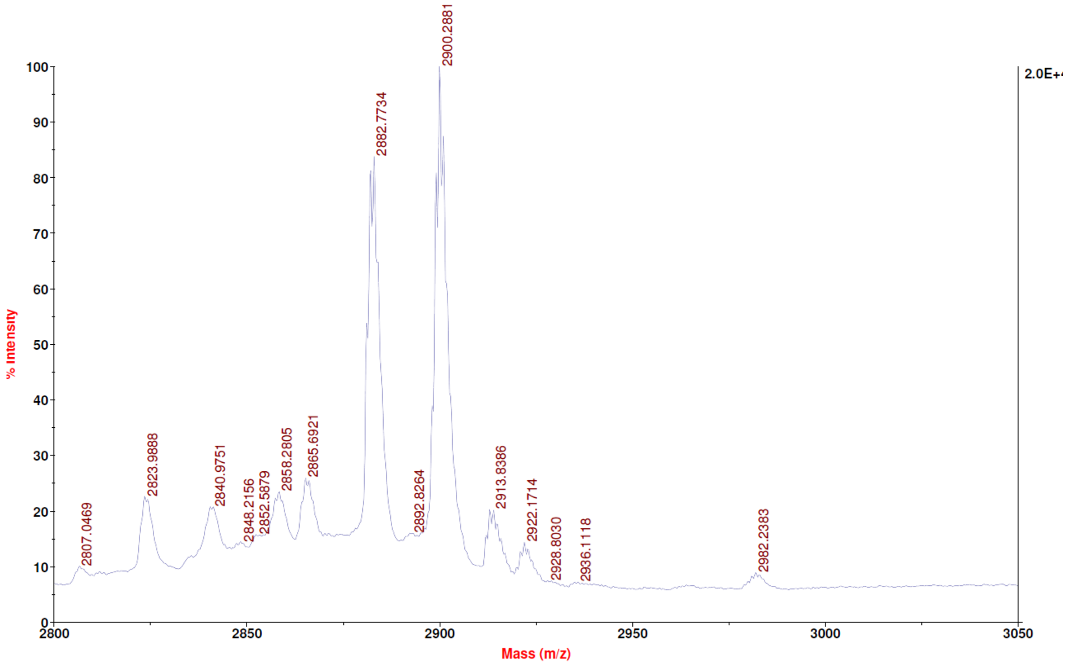


**Figure S5.** *MALDI-TOF MS analysis of orexin B in presence of DEA NONO-Ate in phosphate buffer. Matrix: α-Cyano-4-hydroxycinnamic acid in 9: 1 = MeCN: $H_2O$ + 0.1% TFA. Orexin B [M+H$^+$] =2899 m/z.*

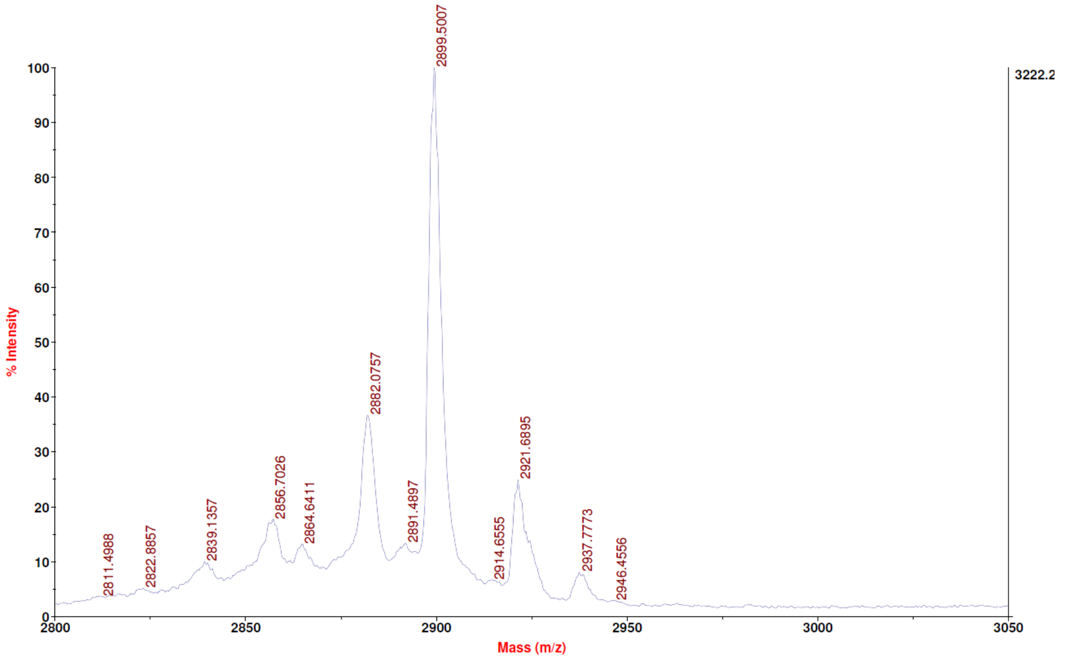


**Figure S6.** *MALDI-TOF MS analysis of orexin B in presence of DEA NONO-Ate in 80% TFE. Matrix: α-Cyano-4-hydroxycinnamic acid in 9: 1 = MeCN: $H_2O$ + 0.1% TFA. Orexin B [M+H$^+$] =2899 m/z.*

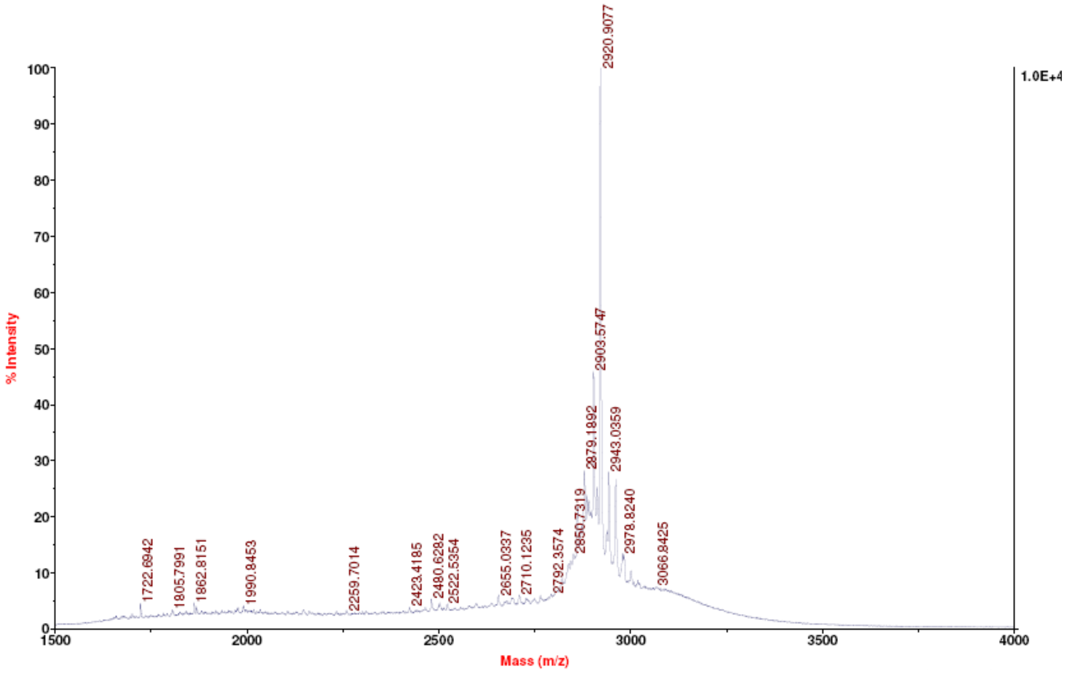


**Figure S7.** *MALDI-TOF MS analysis of orexin B in presence of glyoxal in phosphate buffer. Matrix: α-Cyano-4-hydroxycinnamic acid in 9: 1 = MeCN: $H_2O$ + 0.1% TFA. Orexin B [M+H$^+$] =2899 m/z.*

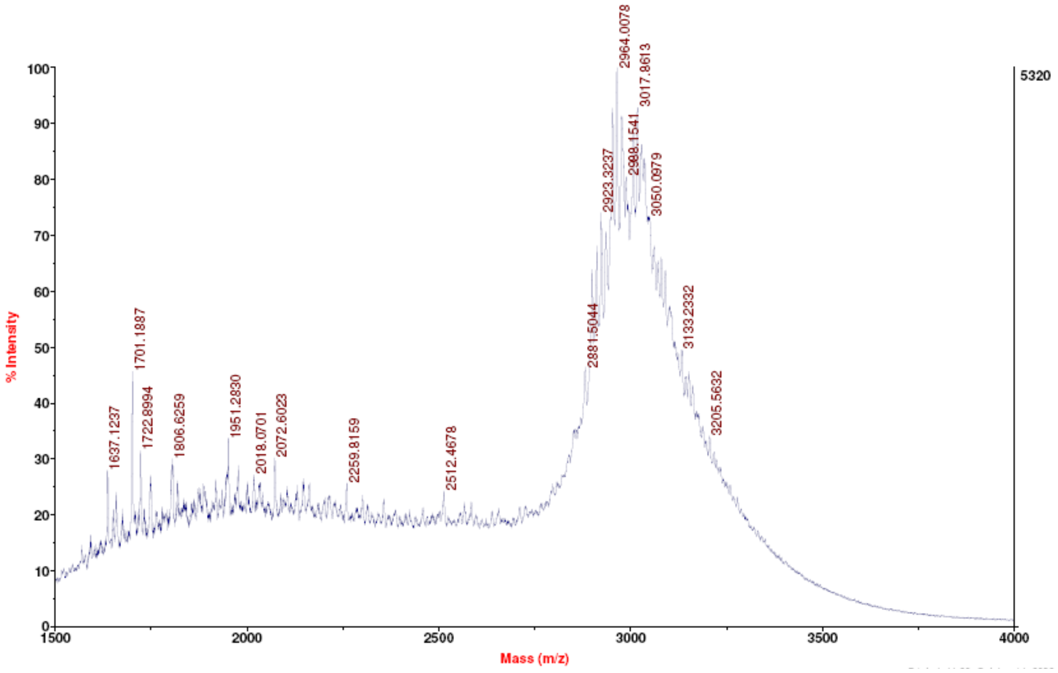


**Figure S8.** *MALDI-TOF MS analysis of orexin B in presence of pyruvic aldehyde in phosphate buffer. Matrix: 2,5-dihydroxybenzoic acid in 9: 1 = MeCN: $H_2O$ + 0.1% TFA. Orexin B [M+H$^+$] =2899 m/z.*

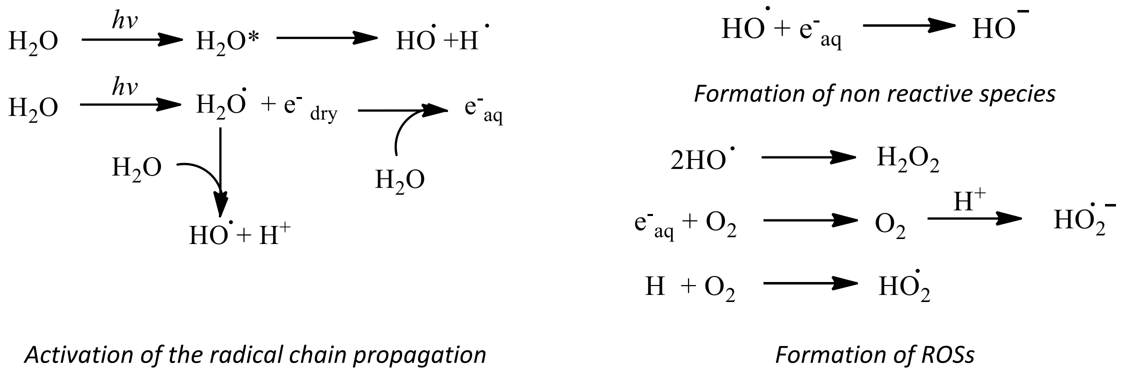


**Scheme S1.** *Mechanism of ROS generation in aqueous medium due to UV irradiation.*

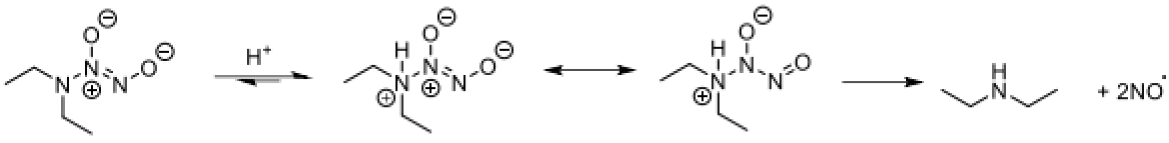

**Scheme S2.** *Mechanism of NO· radical formation due to DEA NONO-Ate.*

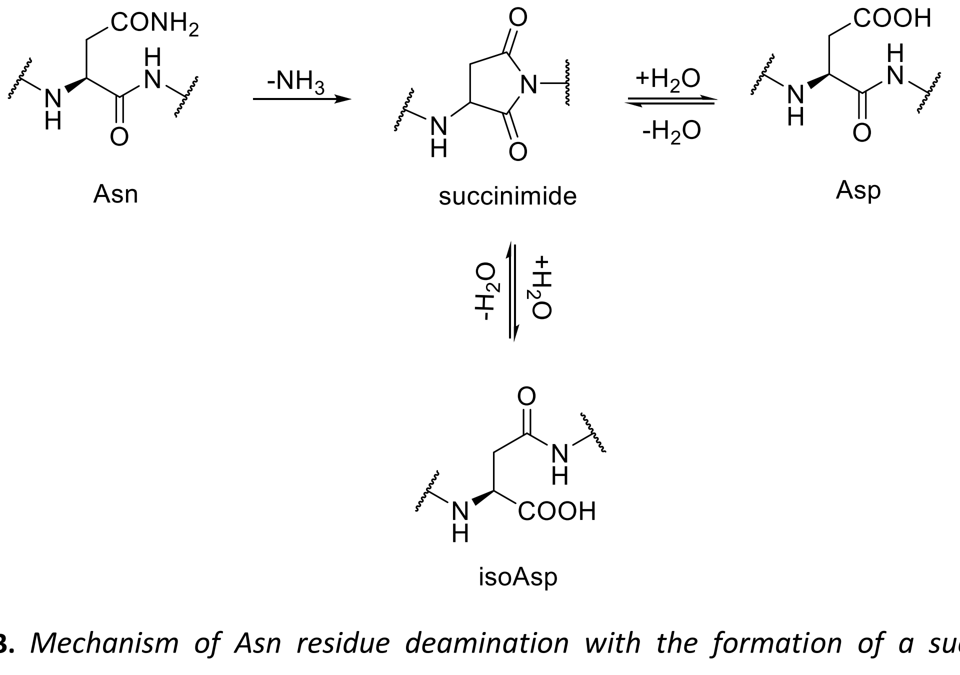


**Scheme S3.** *Mechanism of Asn residue deamination with the formation of a succinimide intermediate.*

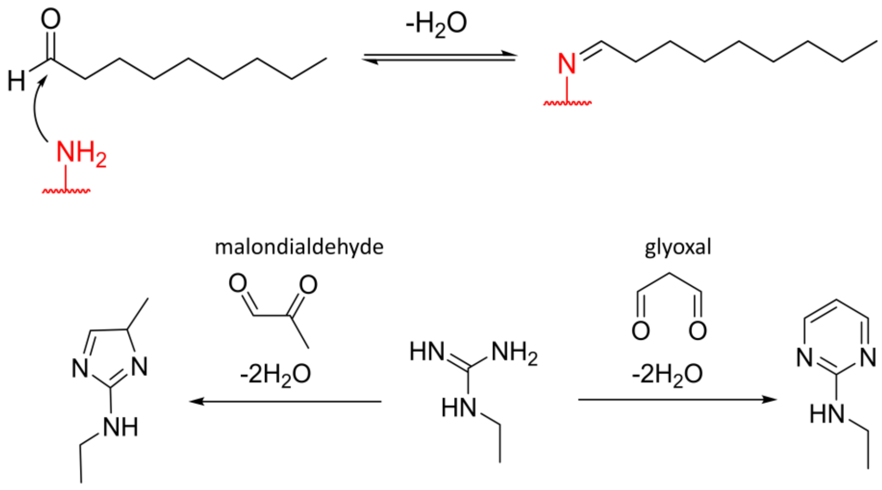


**Scheme S4.** *Mechanism of stable adducts formation among glyoxal or pyruvic aldehyde with amino acid residues.*
